# Association between carotid plaque characteristics and acute cerebral infarction determined by MRI in patients with type 2 diabetes mellitus

**DOI:** 10.1186/s12933-017-0592-9

**Published:** 2017-09-11

**Authors:** Beibei Sun, Xiao Li, Xiaosheng Liu, Xiaoqian Ge, Qing Lu, Xihai Zhao, Jun Pu, Jianrong Xu, Huilin Zhao

**Affiliations:** 1 Department of Radiology, Renji Hospital, School of Medicine, Shanghai Jiao Tong University, 160 Pujian Road, Shanghai, China; 2 Center for Biomedical Imaging Research, Department of Biomedical Engineering, Tsinghua University School of Medicine, Beijing, China; 3 Department of Cardiology, Renji Hospital, School of Medicine, Shanghai Jiao Tong University, 160 Pujian Road, Shanghai, China

**Keywords:** Diabetes mellitus, Acute stroke, Carotid plaque, Lipid-rich necrotic core, MR imaging

## Abstract

**Background:**

Type 2 diabetes mellitus (T2DM) might aggravate the carotid plaque vulnerability, and increase the risk for ischemic stroke. Few studies reported the acute stroke subtype with carotid plaque characteristics in T2DM patients. This study aimed to investigate the association between carotid plaque characteristics and acute cerebral infarct (ACI) lesion features determined by MRI in T2DM patients.

**Methods:**

Patients with acute cerebrovascular syndrome in internal carotid artery territory were recruited. All patients were stratified into T2DM and non-T2DM groups and underwent both carotid and brain MRI scans. Ipsilateral carotid plaque morphological and compositional characteristics, intracranial and extracranial carotid artery stenosis were also determined. Stroke subtype based on the Trial of ORG 10172 in Acute Stroke Treatment classification and ACI lesion patterns were evaluated.

**Results:**

Of the recruited 140 patients, 68 (48.6%) patients had T2DM (mean age 64.16 ± 11.38 years, 40 males). T2DM patients exhibited higher prevalence of carotid type IV–VI lesions, larger plaque burden as well as larger lipid-rich necrotic core (LRNC) compared with non-T2DM patients. Among the patients with carotid LRNC on symptomatic side, more concomitant large perforating artery infarct patterns and larger ACI size in the internal carotid artery territory were found in T2DM group than those in non-T2DM group. Carotid plaque with LRNC% > 22.0% was identified as an independent risk factor for the presence of ACI lesions confined to the carotid territory in T2DM patients, regardless of other risk factors.

**Conclusions:**

This study shows that more concomitant large perforating artery infarct patterns and larger ACI size in the internal carotid artery territory were found in the T2DM patients with ipsilateral carotid LRNC plaque than those in non-T2DM patients. Quantification of the carotid plaque characteristics, particularly the LRNC% by MRI has the potential usefulness for stroke risk stratification.

## Background

Diabetes mellitus is a well-established risk factor for atherosclerosis [1]. Type 2 diabetes mellitus (T2DM) might increase the carotid plaque vulnerability by aggravating inflammation, increasing vasa-vasorum neovascularization, and promoting lipid-core expansion [2]. Although several studies using carotid ultrasound and multidetector CT (MDCT) have reported several ultrasound and CT features of carotid plaques in T2DM patients [3, 4], carotid ultrasound and MDCT could not accurately provide critical tissue characteristics of carotid plaque vulnerability [i.e., lipid-rich necrotic core (LRNC) and intraplaque hemorrhage (IPH)] [5].

MRI can assess both qualitative and quantitative vulnerable characteristics of carotid plaque [6, 7] using multi-contrast vessel wall sequences and acute cerebral infarct (ACI) lesion features on diffusion-weighted imaging (DWI). Few studies have reported the acute stroke subtype with carotid plaque characteristics in T2DM subjects.

Thus, in this study we (1) qualitatively and quantitatively compared the vulnerable characteristics of carotid plaques by MRI in subjects with and without T2DM; and (2) determined the association between the carotid plaque characteristics and ACI lesion features on MRI in T2DM patients with acute stroke.

## Methods

### Study population

Patients with acute cerebrovascular syndrome hospitalized in Shanghai Jiao Tong University Renji Hospital were consecutively recruited from the neurology department between September 2011 and July 2014. The patients with ischemic stroke in the internal carotid artery territory underwent carotid vessel wall and brain MR examinations including diffusion-weighted imaging (DWI) and MR angiography (MRA) within 1 week of symptom onset. The exclusion criteria were as follows: (1) amaurosis fugax; (2) high-risk cardioembolic sources identified by electrocardiogram or transthoracic echocardiography (e.g., atrial fibrillation); (3) previous carotid endarterectomy on the index side or previous neck irradiation; (4) other etiologies, such as vasculitis, moyamoya disease or cancer-related stroke; and (5) contraindications to MRI. The symptomatic carotid artery was defined as being ipsilateral to the acute lesion in the internal carotid artery territory or responsible for the neurological symptoms [8]. The study protocol was approved by the Institutional Review Board of Renji Hospital. Informed consent was obtained from all the subjects.

### Cardiovascular risk factors

Data from medical records including the neurological examination [National Institutes of Health Stroke Scale (NIHSS) score], laboratory analysis [i.e., lipid level, high-sensitivity C-reactive protein (hs-CRP), serum creatinine (Scr), glomerular filtration rate (GFR), hemoglobin A1c (HbA1)], and patient’s baseline information, including age, sex, body mass index (BMI), and cardiovascular risk factors [e.g., T2DM, dyslipidemia, hypertension, current smoking, and a prior transient ischemic attack (TIA)/stroke] were collected upon admission. The patients with T2DM were diagnosed based on their blood glucose levels, i.e., either a fasting plasma glucose (FPG) level of ≥7.0 mmol/l or an OGTT result of ≥11.1 mmol/l. Hypertension was defined as a systolic blood pressure (l) either a fasastolic blood pressure ≥90 mmHg, or current treatment with antihypertensive agents. Dyslipidemia was defined as TC/HDL-C ratio ≥5; measured LDL-C ≥ 3.5 mmol/l; or taking lipid-modifying medications [9]. Smoking status was assessed at the time of the ischemic event, and the patients were dichotomized into two groups: current smoker (recent 3 years) or not a current smoker.

### Classification of stroke subtypes

Stroke subtypes were classified into 5 categories based on etiology, using the TOAST classification: (1) large-artery atherosclerosis, (2) small-artery occlusion, (3) cardioembolism (CE), (4) stroke of other determined etiology, and (5) stroke of undetermined etiology [10]. We modified the definition of large-artery atherosclerosis as with ≥50% stenosis in carotid artery or middle cerebral artery (MCA) on TOF MRA; small vessel occlusion as an ACI lesion diameter of <15 mm and located in a subcortical area on DWI [11].

ACI lesions in the internal carotid artery territory were also allocated to one of the following 6 patterns [12]: single lesion—(1) small perforating artery infarct (PAI) with diameter ≤1.5 cm; (2) large PAI with diameter >1.5 cm; (3) pial infarct; (4) large territorial infarct; (5) borderzone infarct; and (6) multiple lesions.

### MR imaging

All the subjects underwent brain and carotid MRI on a 3.0-T whole-body scanner (Philips Achieva, Best, the Netherlands). A routine MR protocol including diffusion weighted imaging (DWI) and time of flight (TOF) MRA, was used for brain imaging. The imaging parameters of DWI sequence are as follows: repetition time (TR)/echo time (TE) 1598/46 ms, b values = 0.1000 s/mm^2^, matrix of 128 × 128, slice thickness of 6 mm. Three-dimensional (3D) TOF MRA was also acquired for intracranial arteries with the following parameters: TR/TE 23/3.5 ms, flip angle 18°, field of view 199 × 199 mm^2^, matrix 500 × 332, and slice thickness 1.2 mm.

For carotid vessel wall imaging, a previously published multi-contrast protocol was used for plaque detection [3D TOF, T1-weighted, T2-weighted, 3D magnetization-prepared rapid acquisition gradient-echo (MPRAGE) and post-contrast T1-weighted sequences] [13]. Post-contrast T1-weighted images were acquired at about 5 min after contrast agent injection (Magnevist, Bayer Healthcare, Berlin, Germany). The longitudinal coverage of the vessel wall sequences were 32 mm (16 sections). Carotid MRA images were reconstructed from the 3D TOF images. The total acquisition time was approximately 35 min.

### Image analysis

Two trained reviewers (X.Z. and H.Z.; more than 3 years of experience in carotid plaque imaging) interpreted the carotid MR images in the symptomatic side via consensus using a custom-designed software (CASCADE, Seattle, WA, USA) [14]. The reviewers were blinded to the brain MRI scans and clinical information. Image quality rating was assigned using a 4-point scale (1 = poor, 4 = excellent). Slices with image quality <2 were excluded from review. The morphological measurements, including the maximum wall thickness (max WT) and percent wall volume were measured and calculated for each artery. The luminal stenosis of the symptomatic carotid arteries was measured using the NASCET criteria [15]. The presence or absence of carotid plaque component [e.g., LRNC, IPH, calcification, or fibrous cap rupture (FCR)] was identified based on previously published criteria which were validated by histology [7]. Quantitative assessment of the volume of calcification, LRNC and IPH was determined. The percent volume of each component was described as the percent cross-sectional volume of the total plaque.  Carotid atherosclerotic plaque was defined as lesions with presence of any plaque component (e.g., CA, LRNC, FCR or IPH) on MR images. According to the modified American Heart Association (AHA) criteria [6], type IV–V was assigned to plaques characterized by a lipid or necrotic core surrounded by fibrous tissue with possible calcification. Type VI was assigned to complex plaques with a possible FCR, IPH or thrombus.

Brain MR images were evaluated by two experienced radiologists (Q.L. and X.G., >5 years of experience in neuroradiology) with consensus blinded to clinical information and carotid MR images. ACI lesions in the internal carotid artery territory were localized by using DWI, which were defined when lesions had hyperintense on DWI images and hypointense on the apparent diffusion coefficient map. The sum size for ACI lesions in the internal carotid artery territory was recorded on the symptomatic side of each subject. The stenosis of middle cerebral artery (MCA) M1 segment on TOF MRA was also evaluated using the NASCET criteria and graded as <50% diameter stenosis and ≥50%.

### Statistical analysis

Univariate and multivariate logistic regression analyses were performed to assess the risk factors for the presence of LRNC plaques and ACI lesions. The quantitative and categorical data are presented as mean ± SD and percentages, respectively. The continuous variables were compared via the independent-sample *t* test when the data were normally distributed or the Mann–Whitney U test when the data were non-normally distributed. The categorical variables were compared using the Chi square test. A receiver operator curve (ROC) analysis was performed to evaluate the discriminative strength of plaque features for ACI lesion in T2DM patients and non-T2DM patients. The ROC analysis was performed using Medcalc version 11.4.2.0 and the other analyses in this study were performed using R 2.11.0 (R De-velopment Core Team 2010). All of the tests were 2-tailed, and P < 0.05 were considered significant.

## Results

### Baseline characteristics of symptomatic patients with and without DM

During the study period, 512 patients with ischemic stroke in the internal carotid artery territory were consecutively hospitalized at our hospital. A total of 149 patients meeting the inclusion criteria were recruited and successfully performed the carotid and brain MRI for this study. Nine patients were finally excluded because of inadequate MR scan quality. Of the remaining 140 patients, 84 (60%) were male, 81 (57.9%) had hypertension, and 68 (48.6%) had T2DM. The baseline clinical characteristics of T2DM patients and non-T2DM patients are presented in Table 1. T2DM patients had higher Hs-CRP with significant difference (*P* = 0.006) and higher dyslipidemia, Scr and history of stroke at the very edge of significance compared to non-T2DM patients. The presence of hypertension, current smoking and NIHSS score was not significantly different between these two groups (all *P* > 0.05).

**Table 1:** Baseline characteristics in T2DM and non-T2DM patients

	Mean ± SD or n (%)		*P* value
	T2DM (n = 68)	Non-T2DM (n = 72)	
*Risk factors*			
Male	40 (58.8%)	44 (61.1%)	0.842
Age, years	64.16 ± 11.38	62.45 ± 9.73	0.429
BMI, kg/m^2^	24.89 ± 2.68	24.07 ± 2.81	0.151
Hypertension	44 (64.7%)	37 (51.3%)	0.533
Current smoking	39 (57.4%)	44 (61.1%)	0.651
Dyslipidemia	51 (75.0%)	43 (59.7%)	0.054
Scr, µmol/l	79.04 ± 24.65	69.51 ± 16.46	0.056
GFR, ml/min	86.67 ± 30.70	94.59 ± 25.01	0.106
Hs-CRP, mg/l	7.14 ± 6.88	3.85 ± 4.61	0.006
History of stroke/TIA	23 (33.8%)	14 (19.4%)	0.054
NIHSS score	4.48 ± 3.59	4.26 ± 3.43	0.710
*Carotid plaque features*			
Presence of plaque	56 (82.4%)	42 (58.3%)	0.002
AHA type IV–VI	53 (77.9%)	38 (52.8%)	0.002
Luminal stenosis (%)	31.2 ± 33.17	19.42 ± 25.91	0.013
Max WT (mm)	3.22 ± 1.73	2.26 ± 1.17	0.001
Percent wall volume	42.76 ± 11.85	37.78 ± 11.05	0.011
LRNC prevalence	53 (77.9%)	38 (52.8%)	0.002
% volume of LRNC^a,b^	35.35 ± 26.99	15.96 ± 17.28	0.001
IPH or FCR prevalence	9 (13.2%)	6 (8.3%)	0.349
% volume of IPH^a,b^	3.00 ± 9.30	2.21 ± 8.43	0.667
CA prevalence	35 (51.5%)	29 (40.3%)	0.184
% volume of CA^a,b^	5.84 ± 14.63	5.49 ± 8.83	0.891

*T2DM* type 2 diabetes mellitus, *BMI* body mass index, *Scr* serum creatinine, *GFR* glomerular filtration rate, *Hs-CRP* high-sensitivity C-reactive protein, *TIA* transient ischemic attack, *Max WT* maximum wall thickness, *LRNC* lipid-rich necrotic core, *IPH* intraplaque hemorrhage, *FCR* fibrous cap rupture, *CA* calcification

^a^Component% = Corresponding component volume/Plaque volume

^b^Only including those with plaque present

### Comparison of carotid plaque characteristics on symptomatic side between T2DM and non-T2DM patients

**Fig. 1:**
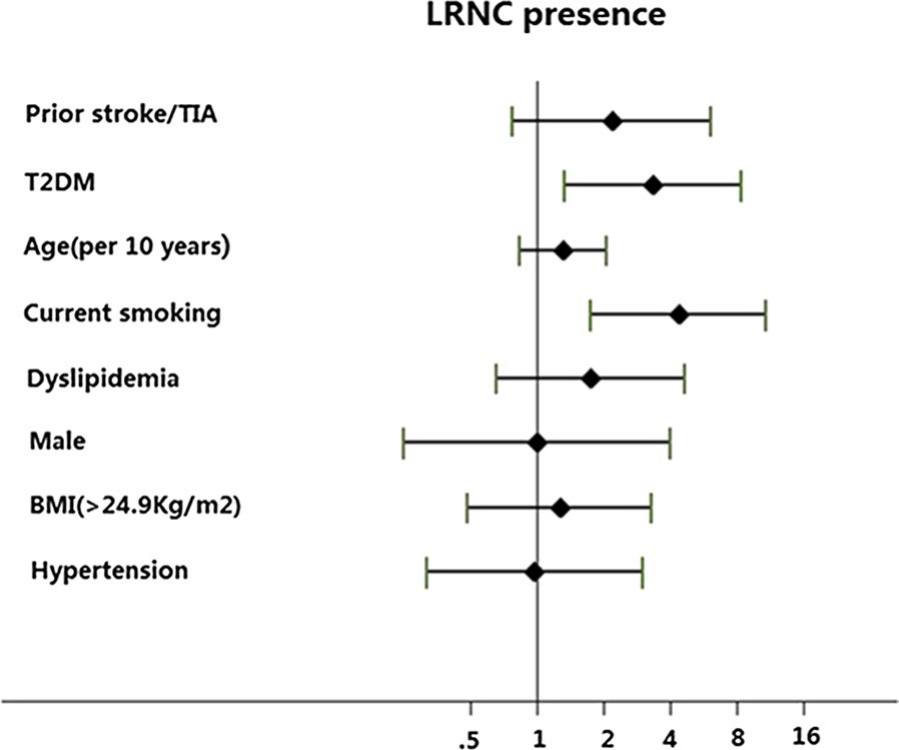
Independent predictors for the presence of carotid LRNC plaques on symptomatic side

The MRI characteristics of the carotid plaques on symptomatic side in patients with and without T2DM are presented in Table 1. The prevalence of carotid plaque and AHA type IV–VI lesions was higher in T2DM patients compared to non-T2DM patients (82.4% vs 58.3%, *P* = 0.002; 77.9% vs 52.8%, *P* = 0.002). Carotid plaque burden were significantly larger in T2DM patients than in non-T2DM patients, as evidenced by higher luminal stenosis (31.20% ± 33.17 vs 19.42% ± 25.91, *P* = 0.013), max WT (3.22 mm  ± 1.73 vs 2.26 mm ± 1.17, *P* = 0.001) and percent wall volume (42.76% ± 11.85 vs 37.78% ± 11.05, *P* = 0.011). A higher prevalence of LRNC and larger LRNC% volume was found in T2DM patients than in non-T2DM patients (77.9% vs 52.8%, *P* = 0.002; 35.35% ± 26.99 vs 15.96% ± 17.28, *P* = 0.001). Furthermore, T2DM was identified as an independent risk factor for the presence of LRNC plaques (OR = 3.35; 95% CIs = 1.33–8.43), regardless of prior stroke/TIA, age (per 10 years) and current smoking (Fig. 1).

### Patients with carotid LRNC plaque on symptomatic side under stroke subtype in T2DM and non-T2DM patients

The characteristics of enrolled patients with carotid LRNC plaque on symptomatic side in T2DM patients and non-T2DM patients are shown in Table 2. Based on the results, patients were grouped according to the TOAST classification. The prevalence of these subtype have no significant difference between T2DM and non-T2DM group. However, T2DM patients with carotid LRNC plaque had more concomitant large PAI and less concomitant small PAI as compared with non-T2DM patients (34.0% vs 13.2%, *P* = 0.024; 13.2% vs 31.6%, *P* = 0.033; respectively).

**Table 2:** Patients with carotid LRNC plaque on symptomatic side under stroke subtype

	T2DM (n = 53)	Non-T2DM (n = 38)	*P* value
*Etiological subtypes*			
Large-artery atherosclerosis	21 (39.6%)	14 (36.8%)	0.788
Small-artery occlusion	20 (37.7%)	21 (55.2%)	0.097
Stroke of other etiology	12 (22.6%)	5 (13.2%)	0.252
*ACI lesion patterns*			
Small PAI (diameter ≤1.5 cm)	7 (13.2%)	12 (31.6%)	0.033
Large PAI (diameter >1.5 cm)	18 (34.0%)	5 (13.2%)	0.024
Pial infarct	0 (0%)	3 (7.9%)	0.069
Large territorial infarct	9 (17.0%)	4 (10.5%)	0.386
Borderzone infarct	1 (1.9%)	0 (0%)	0.582
Multiple lesions	13 (24.5%)	7 (18.4%)	0.488
*ACI lesion size*			
ACI size (ml)^a^	15.45 ± 8.97	9.09 ± 8.64	0.011
ACI size in patients concurrent carotid IPH or FCR (ml)^a^	18.28 ± 6.88	11.51 ± 12.51	0.225
ACI size in patients concurrent carotid ≥50% stenosis (ml)^a^	22.25 ± 5.32	14.18 ± 11.94	0.212
ACI size in patients concurrent MCA ≥50% stenosis (ml)^a^	19.43 ± 7.29	9.45 ± 13.12	0.100

*LRNC* lipid-rich necrotic core, *ACI* acute cerebral infarct, *PAI* large artery atherosclerosis, *MCA* middle cerebral artery, *IPH* intraplaque hemorrhage, *FCR* fibrous cap rupture

^a^Patients with ACI lesions

No significant difference in the prevalence of ACI lesions in the internal carotid artery territory was found in these two groups (90.6% vs. 86.8%, *P* = 0.211). However, T2DM patients had a larger ACI size than non-T2DM patients (15.45 ml ± 8.97 vs. 9.09 ml ± 8.64, *P* = 0.011). In contrast, no significant difference in ACI size was found in patients concurrent carotid or MCA ≥ 50% stenosis, and concurrent carotid IPH or FCR between T2DM and non-T2DM group.

**Fig. 2:**
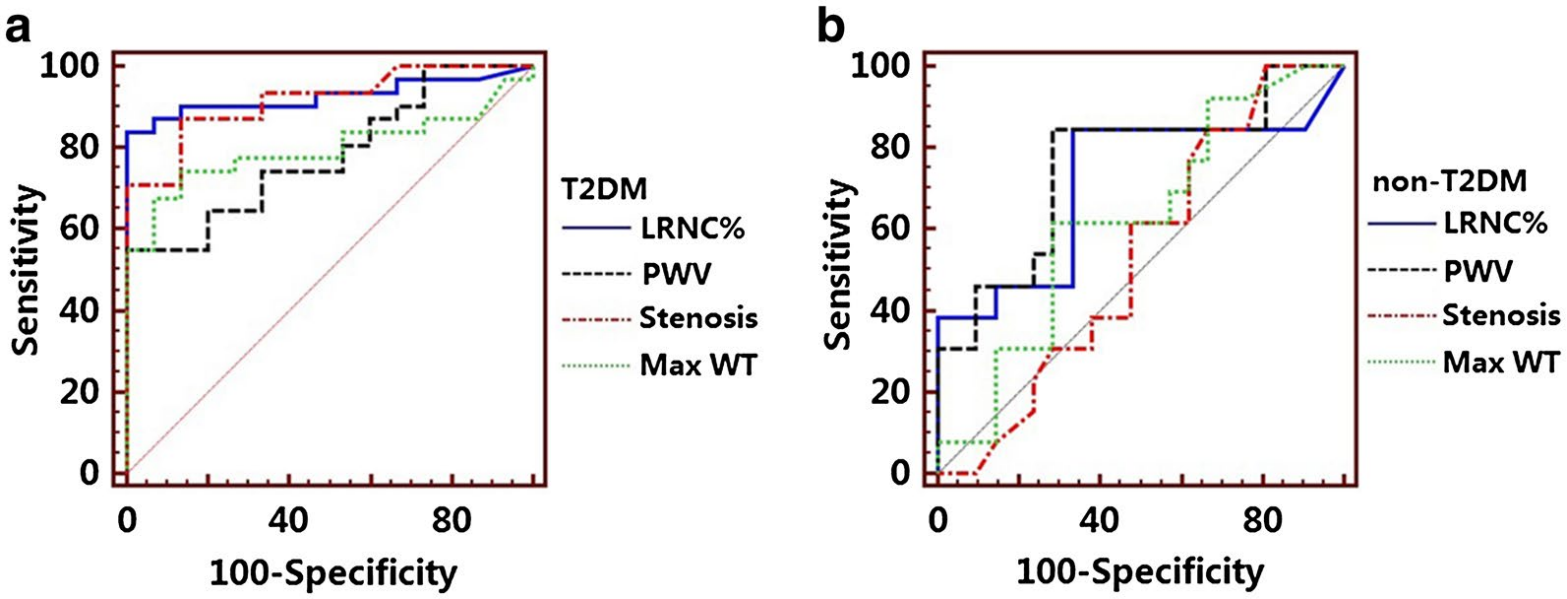
Optimal threshold of carotid plaque features to predict the presence of ACI in ICA region in T2DM subjects (**a**) and non-T2DM subjects (**b**). *T2DM* type 2 diabetes mellitus, *LRNC%* % volume of LRNC, *PWV* Percent wall volume, *Max WT* maximum wall thickness

In T2DM patients, ROC analysis showed that 22.0% was the optimal threshold of LRNC% to predict the presence of ACI in the internal carotid artery territory (Fig. 2a). The use of this value yielded a sensitivity of 83.87% and specificity of 97.8% (AUC = 0.927). Meanwhile, the ROC curve showed that 11.6% was the optimal cutoff value of stenosis level to predict the presence of ACI (AUC = 0.916, sensitivity, 87.1%; specificity, 86.7%) (Fig. 2a).

In contrast, 4.32% was shown to be the optimal threshold of LRNC% to predict the presence of ACI in the internal carotid artery territory in non-T2DM patients. The use of this value yielded a sensitivity of 84.62% and specificity of 66.67% (AUC = 0.714) (Fig. 2b).

### Association between carotid LRNC plaques and ACI features in T2DM patients

**Fig. 3:**
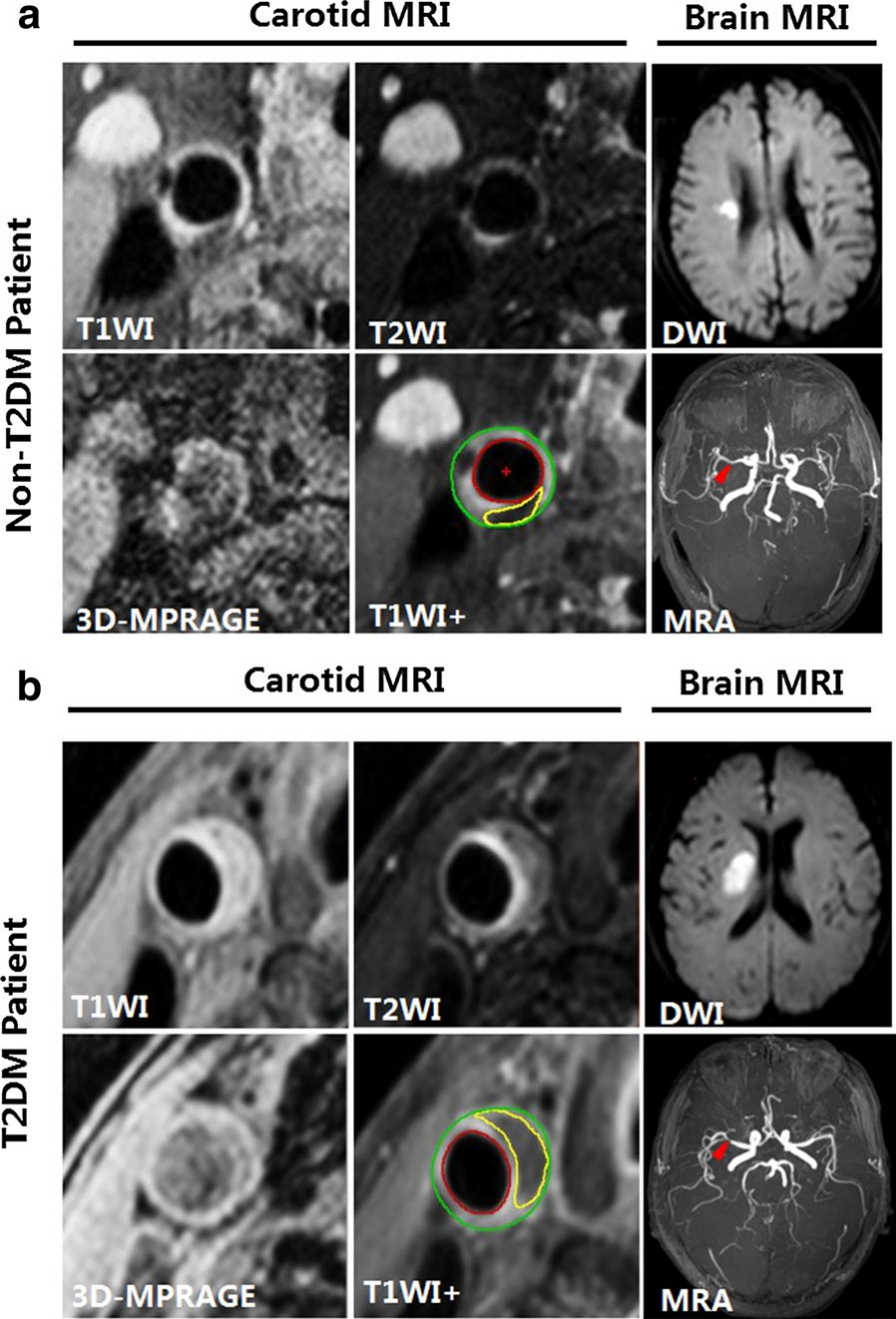
Representative MR images of a T2DM and a non-T2DM subject. **a** An atherosclerotic plaque with small LRNC (*yellow outline*) is detected in the right carotid artery of a non-T2DM patient: iso-intensity on T1WI and T2WI; no enhancement in T1WI+; hypo-intensity on MP-RAGE image. Cerebral DWI demonstrates a small perforating artery infarct (hyper-intensity) at right hemisphere. Brain MRA demonstrates right MCA M1 segment with mild stenosis (*red arrow*). **b** An atherosclerotic plaque with large LRNC (*yellow outline*) with mild to moderate stenosis is detected in the right carotid artery of a T2DM patient. LRNC appears as iso-intensity on T1WI; hypo-intensity on corresponding T2WI; no enhancement in T1WI+; hypo-intensity on MP-RAGE image. Cerebral DWI demonstrates large volume of ACI (hyperintensity) at right hemisphere. Brain MRA demonstrates right MCA M1 segment with moderate stenosis (*red arrow*). *ACI* acute cerebral infarct, *LRNC* lipid-rich necrotic core, *WI* weighted image, *T1WI+* contrast enhanced T1-weighted image, *MCA* middle cerebral artery

For symptomatic carotid arteries with plaque, significant association of carotid LRNC% > 22.0% and the ACI presence in internal carotid artery territory was found during univariate analysis (OR = 12.41; 95% CIs = 1.42–108.31, *P* = 0.023, Table 3). After adjusting for significant demographic factors, the association remained statistically significant (multivariate adjusted OR = 12.5; 95% CIs = 2.81–55.43 *P* = 0.001). Figure 3 are examples showing subjects with carotid LRNC and ipsilateral ACI lesions.

**Table 3:** Univariate and multivariate logistic regression of vascular bed variables for the presence of ACI lesions in the internal carotid artery territory in T2DM patients with carotid plaque (*n* = 56)

	Univariate			Multivariate^a^		
	OR	95% CI	*P* value	OR	95% CI	*P* value
Carotid stenosis ≥50%	2.93	0.25–35.01	0.396	3.24	0.33–32.39	0.316
MCA stenosis ≥50%	1.62	0.11–24.82	0.728	1.56	0.13–19.37	0.728
IPH or rupture	0.84	0.06–11.85	0.899	0.84	0.06–11.85	0.899
LRNC% > 22%	12.41	1.42–108.31	0.023	12.50	2.81–55.43	0.001

*T2DM* type 2 diabetes mellitus, *MCA* middle cerebral artery, *IPH* intraplaque hemorrhage, *FCR* fibrous cap rupture, *LRNC%* % volume of lipid-rich necrotic core

^a^Adjusted for significant demographic factors, gender, age, BMI, hypertension, current smoking, dyslipidemia, history of stroke/TIA

## Discussion

The present study using high-resolution MRI to access the carotid atherosclerotic plaque characteristics, intracranial arterial stenosis and ACI lesion features in symptomatic subjects with non-cardioembolism stroke. We found that: (1) T2DM patients exhibited higher prevalence of carotid type IV–VI lesions, larger plaque burden as well as larger LRNC plaques compared with non-T2DM patients; (2) among the patients with carotid LRNC plaque on symptomatic side, more concomitant large PAI patterns and larger ACI size in the internal carotid artery territory were found in T2DM patients than those in non-T2DM patients; (3) carotid plaque with LRNC% > 22.0% was identified as an independent risk factor of the presence of ACI lesions confined to the carotid territory in T2DM patients, regardless for other risk factors. Our results suggest that MRI-identified vulnerable features, especially large LRNC%, are closely related to ACI patterns and size in T2DM patients suffering an acute stroke.

The present study showed that T2DM patients with acute stroke exhibit higher prevalence of carotid type IV–VI lesions and larger plaque burden than non-T2DM patients. Furthermore, we found that T2DM was independently associated with MRI-identified LRNC plaques in the symptomatic carotid arteries, regardless of other risk factors. The above results of our study are consistent with observations from the study of Esposito et al. which showed that T2DM was associated with AHA IV–VI lesion type plaques in moderate to severe carotid artery stenosis without stroke [16]. Alonso’s study [17] showed that diabetic retinopathy in T2DM patients without cardiovascular disease and with normal renal function is associated with a higher atherosclerotic burden in the carotid arteries. Postmortem studies have demonstrated that coronary artery lesions from T2DM subjects had larger mean necrotic cores than non-T2DM subjects [18, 19]. Pre-clinical studies showed that hyperglycemia and some of its end products can cause severe inflammatory infiltration and larger LRNCs in diabetic atheroma [19]. Huang et al. [20] and our previous study [21] found a quantitative relationship between the HbA1c levels and plaque features in ultrasonic or MR images of atherosclerotic patients. These results support the findings that T2DM may have an adverse effect on carotid plaque vulnerability. In contrast, some other studies, particularly the Rotterdam Study [22] and AIM-HIGH Study [23], have shown that diabetes mellitus is not associated with a higher risk of the presence of LRNC. These disparate conclusions might be due to differences in study designs, cohorts or analytic approaches. The patients recruited in our study were with acute cerebrovascular syndrome, whereas the Rotterdam Study participants were included mainly by carotid wall thickening ≥2.5 mm, and the subjects in AIM-HIGH Study were enrolled with more females, more metabolic syndrome and fewer DM. Additionally, there were significant differences in racial distribution among these MRI sub-studies. Previous studies revealed that the composition and morphology of carotid atherosclerotic lesions were found to be different between ethno-racial groups [11, 24] and the status of metabolic level [25].

In this study, we accessed the association of carotid plaque characteristics with ACI lesions confined to the carotid territory in patients with and without T2DM. We used TOAST classification and the pattern of ACI lesions on DWI help to reveal the mechanism of the cerebral ischemic events [12]. The prevalence of TOAST subtypes had no significant difference between T2DM and non-T2DM patients. However, we found that T2DM patients with carotid LRNC plaques had concomitant more large PAI and less small PAI patterns as compared with non-T2DM patients on the symptomatic side. Previous studies [17, 26–28] have shown that T2DM have unequivocal association with the increased risk for ischemic stroke or worsened outcome following stroke. Chung et al. [11] studied 2702 acute ischemic stroke Asian patients, and found that large-artery atherosclerosis was the leading TOAST subtype in border zone infarction (89.9%) as well as internal carotid territory infarction (51.5%). Large-artery atherosclerosis and small-artery occlusion were the two most common subtypes (38.5 and 22.8%) in single internal carotid territory infarction. Lee et al. [29] revealed that perforating artery infarcts, whether single or occurring in addition to pial or border-zone infarcts, were lesion patterns specific for MCA disease. Multiple ACI lesions are thought to be markers of embolism. Diabetes promotes inflammation infiltration and lipid-core expansion, induced large proportion of LRNC in diabetic atheromas, which are associated with surface disruption and small thrombus formation [30]. It should be noted that the low prevalence of IPH or FCR (only 13.2% in T2DM patients) and mild to moderate luminal stenosis (31.2 ± 33.17% in T2DM patients) were found on the symptomatic carotid arteries in our study, suggesting that the carotid plaques identified in our study were mostly asymptomatic lesions [31]. Xu et al. [32] found that co-existing intracranial and extracranial carotid artery plaques were prevalent in symptomatic patients and the number of co-existing plaques was independently associated with the risk of recurrent stroke.

In our study, ROC analysis showed that 22.0% was the optimal threshold of LRNC% to predict the presence of ACI, yielded a sensitivity of 83.87% and specificity of 97.8% in T2DM patients. Multivariate analysis demonstrated that carotid plaque LRNC% > 22.0% was an independent risk factor for ACI presence confined to the corresponding territory after adjustment of clinical demographic factors. Our findings were in accordance with several carotid ultrasound studies observing an association between the presence of echolucent plaques, an ultrasound signature for unstable plaque, and increased risk of ischemic cerebrovascular events in T2DM patients [33–35]. Meanwhile, this study extended our previous findings which demonstrated a close relationship between higher HbA1c level and ACI severity in stroke patients [21]. An explanation for the association in our findings may be that atherosclerosis is a systemic process. There is evidence that the composition and clinical consequences of plaques at different locations within an individual are similar, and DM promotes inflammation infiltration and lipid-core expansion which might exacerbate the severity of ACI. The results of our study demonstrate that quantification of the carotid plaque characteristics, particularly the LRNC% by MRI may be useful to stratify risk for patients with low-grade carotid stenosis.

Limitations of this study should be noted. First, this was a retrospective single-center study of stroke patients. The selection bias may exist since this study was intended for patients with cerebral infarction, and the generality of the study results is limited. A prospective population-based study should be performed to verify the predictive value. Second, we performed the analyses based on a “yes or no” diagnosis of T2DM upon admission and did not determine the history or glycemic control of diabetes from these patients. Third, because of the generally long acquisition time (>30 min) and limited coverage (<40 mm centered on the carotid bifurcation) of the multi-contrast MR sequences, it was difficult to obtain sufficient cooperation from the patients with severe symptoms. This limited the detection of atherosclerotic lesions occurring in more distal or more proximal segments. Recently, fast 3D multi-contrast vessel wall techniques were proposed for joint detection of intra- and extracranial artery plaques. These techniques may be useful to capture the criminal plaque [36, 37]. In addition, diagnostic modality for noninvasively evaluating cerebral hemodynamics is useful to explore the mechanisms behind acute cerebrovascular syndrome and to determine the ideal therapies [38].

## Conclusions

This study shows that T2DM patients with ipsilateral carotid LRNC plaque exhibited more concomitant large PAI patterns and larger ACI size confined to carotid artery territory than non-T2DM patients. Carotid plaque with LRNC% > 22.0% was identified as an independent risk factor of the presence of ACI lesions in T2DM patients regardless of other risk factors. Our results indicate that quantification of the carotid plaque characteristics, particularly the LRNC% by MRI has the potential usefulness for stroke risk stratification.

## Abbreviations

ACI: acute cerebral infarct
BMI: body mass index
CA: calcification
DWI: diffusion weighted imaging
FCR: fibrous cap rupture
FPG: fasting plasma glucose
GFR: glomerular filtration rate
HbA1c: hemoglobin A1c
HDL-C: high-density lipoprotein cholesterol
Hs-CRP: high-sensitivity C-reactive protein
IPH: intraplaque hemorrhage
ICA: internal carotid artery
LDL-C: low-density lipoprotein cholesterol
LRNC: lipid-rich necrotic core
Max WT: maximum wall thickness
MCA: middle cerebral artery
PAI: perforating artery infarct
Scr: serum creatinine
TC: total cholesterol
T2DM: type 2 diabetes mellitus
TG: triglyceride
TIA: transient ischemic attack
3D TOF: three-dimensional time-of-flight
3D MPRAGE: three-dimensional magnetization-prepared rapid acquisition gradient-echo
